# LncRNA NNT-AS1 regulates proliferation, ECM accumulation and inflammation of human mesangial cells induced by high glucose through miR-214-5p/smad4

**DOI:** 10.1186/s12882-021-02580-y

**Published:** 2021-11-06

**Authors:** Zhuang Geng, Xiang Wang, Shiyuan Hao, Bingzi Dong, Yajing Huang, Yangang Wang, Lili Xu

**Affiliations:** 1 Department of Endocrinology and Metabolism, The Affiliated Hospital of Qingdao University, Qingdao, Shandong 266000 P.R. China; 2 Department of Anesthesiology of the Affiliated Hospital of Qingdao University, Qingdao, Shandong 266000 P.R. China

**Keywords:** NNT-AS1, Extracellular matrix, Human mesangial cells, miR-214-5p, smad4, Diabetic nephropathy

## Abstract

**Background:**

LncRNA NNT-AS1 (NNT-AS1) has been extensively studied as the causative agent in propagation and progression of lung and bladder cancers, and cholangiocarcinoma. However, its significance in proliferation and inflammation of diabetic nephropathy is enigmatic. This study focuses on the molecular mechanisms followed by NNT-AS1 to establish diabetic nephropathy (DN) and its potential miRNA target.

**Methods:**

Bioinformatics analysis to identify potential miRNA target of NNT-AS1 and smad4 transcription factor was conducted using LncBase and TargetScan, and was subsequently confirmed by luciferase reporter assay. Relative quantitative expression of NNT-AS1 in human glomerular mesangial cells (HGMCs) was detected through quantitative real-time PCR and WB analysis. Cell proliferation was detected through CCK-8 assay, whereas, ELISA was conducted to evaluate the expression of inflammatory cytokines. Following this, relative expression of miR-214-5p and smad4 were confirmed through qRT-PCR and western blot analysis.

**Results:**

Results from the experiments manifested up-regulated levels of NNT-AS1 and smad4 in the blood samples of DN patients as well as in HGMCs, whereas, downregulated levels of miR-214-5p were measured in the HGMCs suggesting the negative correlation between NNT-AS1 and miR-214-5p. Potential binding sites of NNT-AS1 showed miR-214-5p as its direct target and NNT-AS1 as potential absorber for this microRNA, in turn increasing the expression of transcription factor smad4.

**Conclusion:**

The data suggests that NNT-AS1 can be positively used as a potential biomarker and indicator of DN and causes extracellular matrix (ECM) accumulation and inflammation of human mesangial cells.

**Supplementary Information:**

The online version contains supplementary material available at 10.1186/s12882-021-02580-y.

## Highlights

1. Up-regulated levels of NNT-AS1 and smad4 in DN samples and HGMCs was manifested.

2. Potential binding sites of NNT-AS1 showed miR-214-5p as its direct target and NNT-AS1 as potential absorber for this microRNA, in turn increasing the expression of transcription factor smad4.

3. The results manifested that knocking-down NNT-AS1 can significantly decrease the inflammation and progression of DN and hence can be used as a potential therapeutic target.

**Supplementary Information:**

The online version contains supplementary material available at 10.1186/s12882-021-02580-y.

## Background

Diabetes mellitus is among the most notorious group of diseases causing complications and malfunctioning of kidneys leading to chronic renal failure [1–3]. Renal failure is most commonly characterized by diabetic nephropathy (DN) which accumulates the extracellular matrix in tubulointerstitial and glomerular regions of the kidney and also thickens the vasculature of the renal region [4]. Besides being the major reason for kidney failure, DN also increases the chances of cardiovascular diseases and is said to affect one-third patients with diabetes mellitus [5]. Moreover, literature reports a substantial global increase in diabetes patients every year and hence, the risk of developing DN is also mounting. Pathophysiological studies suggest the interference of hemodynamic and metabolic factors with normal signalling pathways and cause disease progression [6]. For instance, glucose-dependent pathways, vasoactive hormones, and their receptors directly influence renal functioning due to diabetes and gradually lead to DN [7]. The disease is usually characterized by a gradual decrease in filtration capacity of the glomerular capsule, urea with a high concentration of albumin with up to > 300 mg/day, and constantly elevated blood pressure [8].

In addition to these mutated metabolic pathways and signalling molecules, accumulating literature has suggested the role of non-coding RNAs in development and progression of renal malfunctioning. Showing the structural resemblance to mRNAs, lncRNAs can interfere molecular processes by binding different transcription factors, localization of cellular proteins to specific sites, facilitating the intermolecular interaction of various components, and by differential expression of tissue-specific genes [9, 10]. Also, ncRNAs can influence post-transcriptional modifications by binding to several miRNAs and hence stop their binding with their respective mRNAs [11]. However, the fact that lncRNAs can act as transcriptional activators is still enigmatic and needs to be deciphered. For instance, the role of lncRNAs as direct regulators and oncogenes in carcinomas have been well explored, nonetheless, detailed functional insights into the role of lncRNAs as influencers of DN still needs to be investigated. Such as GM6135, lnc-MGC, lncRNA NR_033515, MALAT-1, NEAT1, PVT1, CYP4B1-PS1-001 have been shown to target mesangial cells and causing extracellular matrix accumulation, mesangial cells proliferation, and DN changes [12–15].

Nevertheless, the significance of NNT-AS1 needs a thorough understanding of the development of DN. NNT-AS1 has been shown its association with cholangiocarcinoma, lung cancer, bladder cancer and other tumours [16–18]. It has also been well reported that NNT-AS1 is greatly upregulated in DN, however, the precise mechanism that how it interferes in renal functioning needs improved understanding. Therefore, this study has been particularly designed to signify the proliferative, inflammatory potential of NNT-AS1 of human mesangial cells. This will allow enhanced understanding of lncRNAs targets, mechanisms, and regulatory roles in DN and how they can act as biomarkers and potential agents for treatment.

## Materials & methods

### Software used for computational analysis

LncBASE (https://carolina.imis.athena-innovation.gr/diana_tools/web/index.php?r=lncbasev2%2Findex-predicted) was used to identify the miRNA target of NNT-AS1 and downstream target gene of miRNA. Whereas, biological targets for miR1179 were validated through TargetScan (http://www.targetscan.org/vert_72/).

### Patient’s tissue samples & ethical approval

The following research was performed after the approval of the local ethics committee of The Affiliated Hospital of Qingdao University and performed in accordance with the Declaration of Helsinki. Word written informed consent was also obtained from all subjects to be included in the research. In total, 22 subjects with diabetic nephropathy (DN) and 20 normal healthy individuals were recruited as participants for the study.

### Cell lines used in the analysis

The HGMCs were used for the analysis and these lines were procured from the Cell Bank of Chinese Academy of Science (Shanghai) and subjected to resuspension according to the standard method [19]. Endothelial basal medium containing hydrocortisone, epidermal growth factor, antibiotic gentamycin, bovine brain extract, and 10% FBS (R&D Systems, USA) was used to culture cell lines at 37 °C, 95% humidity, and 5% CO_2_ (Thermo Scientific, USA). HGMC were divided into two groups based on the concentration of glucose fed. HGMC fed with 25 mmol/L glucose were termed as high glucose (HG) group whereas, low glucose (LG) group was given 5.5 mmol/L glucose in the period of 24 h.

### Extraction of RNA & qRT-PCR for quantitative gene expression

Total tissue RNA was retrieved with Trizol reagent (cat. A33250, Invitrogen) focusing the manufacturer’s guidelines. RNA was quantified with a spectrophotometer (Thermo Scientific). The cDNA was synthesized by using the cDNA Synthesis Kit (cat. 11117831001, Roche) according to the reference guidebook. A qScript One-Step RT-qPCR kit (95057-050, Quanta Bio, USA) was used to perform qRT-PCR in PCR system (Applied Biosystems). Primer set for NNT-AS1: F-5′-CTGGAATCCCTGCTACTCAGGA-3′; R-5′-GCCATGTGATATGCCTGCTC-3′; miR-214-5p: 5′-TGCCTGTCTACACTTGCTGTGC-3′, R- 5′-GGTGCAGGGTCCGAGGTAT-3′, smad4: F- 5′-CCTTCAAGCTGCCCTATTGTTACT-3′, R- 5′- ACATTCCAACTGCACACCTTTG-3′, GAPDH F-5’CCCACTCCTCCACCTTTGAC-3′, R-5’CATACCAGGAAATGAGCTTGACAA-3′, and U-6: 5′-GCTTCGGCAGCACATATACT-3′, and reverse 5′- GTGCAGGGTCCGAGGTATTC-3′ were used according to reference publication. GAPDH and U6 were reference genes and used as internal controls following the analysis of fold changes of quantitative gene expression through the 2 ^-ΔΔCT^ calculation method [20].

### Cell transfection assay

HGMC were transfected to create knockouts as per given manufacturer’s instructions. Transfection was performed by culturing HGMC in 96-well plate with 1 × 10^4^ cells per well. Small interfering RNAs, i.e., si-NNT-AS1#1, si-NNT-AS1#2, si-NC, miR-214-5p inhibitor, and OE-smad4 were procured from Gene Pharma (China). Standard transfection was carried out in pcDNA3.1 using Lipofectamine 3000 reagent (cat. L3000075, Invitrogen, USA) according to the user manual provided and transfection efficiency was measured by qRT–PCR [20].

### Calculation of cell proliferation by cell counting kit 8 (CCK-8) bioassay

HGMC proliferative ability was confirmed with CCK-8 kit (cat. 96,992, Sigma-Aldrich) by following the manufacturer’s guidebook. HGMC were lodged into 96-well plate with 3 × 10^3^ cells per well followed by incubation at 37 °C for 0 h, 24 h, 48 h, 72 h, and 96 h. Following the protocol, 10 μL of CCK-8 proliferation reagent (Sigma-Aldrich) was supplemented to every well subsequently at each time point and incubation at 37 °C for 1 h was carried out. Optical densities of cells were taken at 450 nm with a microplate reader (Becton, Dickinson and Company, USA) [21].

### Luciferase gene reporter assay

The luciferase assay was conducted as narrated by Muramatsu et al. [22] was used to detect luciferase activity. HGMC cells in the concentration of 5 × 10^4^ cells/well were loaded into the 96-well assay plate and co-transfected with pMIR-construct (comprising WT or MUT of NNT-AS1 and pRL-TK *Renilla*) in the presence of Lipofectamine 3000 reagent (cat. L3000075, Invitrogen, USA). Following 24 h incubation, Luciferase Reporter System (Promega, U.S.A.) was used to assess pMIR-luciferase activity, following standard protocol.

### Western blot (WB) analysis

Total proteins were isolated with RIPA buffer followed by the detection of protein’s quality with BCA protein assay kit (cat. P0012S, Beyotime, China). Following quantification, the lysates were subjected to SDS-PAGE analysis and subsequently shifted to Polyvinylidene fluoride (PVDF; Sigma-Aldrich). Next, following incubation at 4 °C for 24 h with the addition of primary antibodies Collagen type I (cat. 81375SF, CST), Collagen type IV (cat. 66887S, CST), Fibronectin (cat. ab2413), GAPDH (cat. ab8245), SMAD4 (cat. ab40759) (1:1000 dilution, Abcam) and washed with tris-buffer saline (cat. SIG-32391, Tocris Bioscience). Following the manufacturer’s instructions, membranes were shifted for second incubation for 1 h with secondary antibodies (cat. ab7090, 1:1000 dilution, Abcam). Results were visualized with chemiluminescence western blotting kit (Global Life Sciences Solutions, USA).

### Enzyme-linked immunosorbent assay to detect inflammatory proteins

ELISA was performed to quantify the inflammatory proteins including TNF-α, IL-1β and IL-6 by ELISA kit (cat. E-EL-H0109c, E-EL-H0149c, E-EL-H0192c, Elascience, China). The cells were cultured in 10% FBS for 48 h and shifted to serum-free medium. Following this, cell-free supernatants were subjected to ELISA analysis and the optical density of cells was measured at 450 nm with 800 TS Absorbance Reader (BioTek, USA).

### RNA immunoprecipitation (RIP) assay

The RIP-RT-qPCR assay was performed to quantify the RNA by using Magna RIP RNA-Binding Kit (cat. 17-700, Merck). HGMC cell lysate was prepared with RIP buffer following incubation with anti-Ago2 and anti-IgG. Following immunoprecipitation, purified RNAs were subjected to reverse-transcription which was confirmed by RT-qPCR analysis [23].

### RNA pull-down assay

To detect RNA pull-down, commercially synthesized NNT-AS1 was used as a probe. First of all, NNT-AS1 DNA sequence was amplified by using T7- primer and the resulting product was subsequently cloned into pCR8 plasmid. Single digestion with NotI restriction enzyme was carried out to linearize the ligated pCR8 plasmid. T7 RNA polymerase and RNA labelling mixture of biotin (cat. Y0001978, Roche Diagnostics) were used to reverse-transcribe the Biotin-labeled RNAs. The reaction mixture was treated with DNase I (RNase-free) (cat. 89,836, ThermoScientific, USA), before RNA purification by RNeasy Plus Mini Kit (Qiagen, USA). The extracted RNA was further used for qRT-PCR.

### Statistical analysis

Data from the experiments were subjected to statistical analysis using GraphPad Prism (GraphPad, USA) and SPSS 20.0 (IBM Corp. USA). Differences within the groups and multiple groups were analyzed through Tukey’s post-hoc test for Student’s t-test and ANOVA. The correlation was determined through Pearson’s correlation analysis. All experiments were performed in accordance with relevant guidelines and regulations.

## Results

### NNT-AS1 expression and HG-induced HGMC

**Fig. 1:**
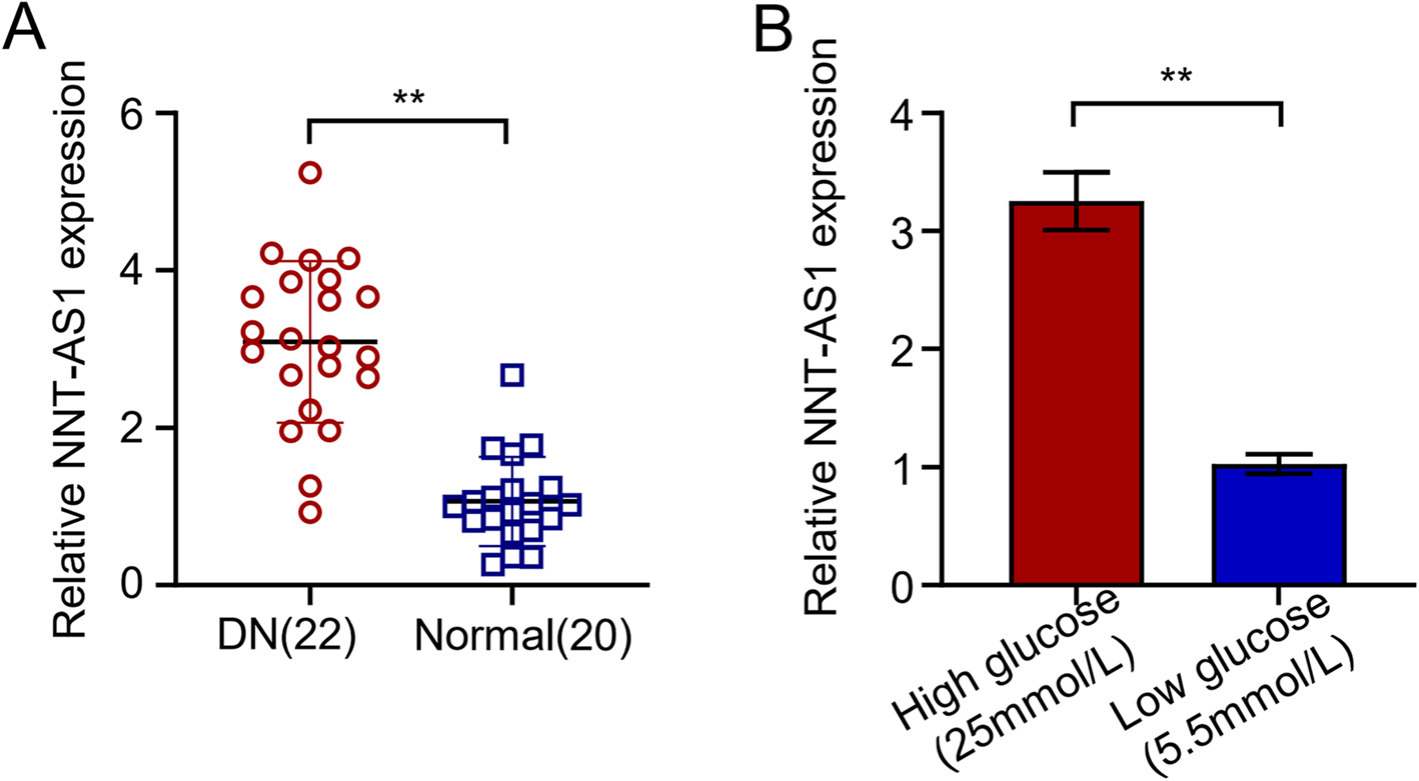
The expression of NNT-AS1 in diabetic nephropathy patients and high glucose induced patients was moderate and high. **A** qRT-PCR indicating NNT-AS1 expression levels in 22 DN and 20 normal patients, **B** qRT-PCR indicating NNT-AS1 expression levels in high glucose induced HGMCs and low glucose group

The expression of NNT-ASI was significantly increased about 2.9 times higher in the blood samples of 22 DN patients than the normal healthy individuals, confirming its higher incidence and a potential biomarker for DN (*P* < 0.01; Fig. 1A). qRT-PCR analysis of HGMC showed considerably higher (about 3.2 times) expression of NNT-AS1 in 25 mmol/L high glucose (HG) group as compared to 5.5 mmol/L low glucose (LG) group and statistically significant differences were observed (*P* < 0.01; Fig. 1B).

### NNT-AS1 serves as a regulator and causes ECM accumulation, HG-induced proliferation, and inflammation in human mesangial cells

**Fig. 2:**
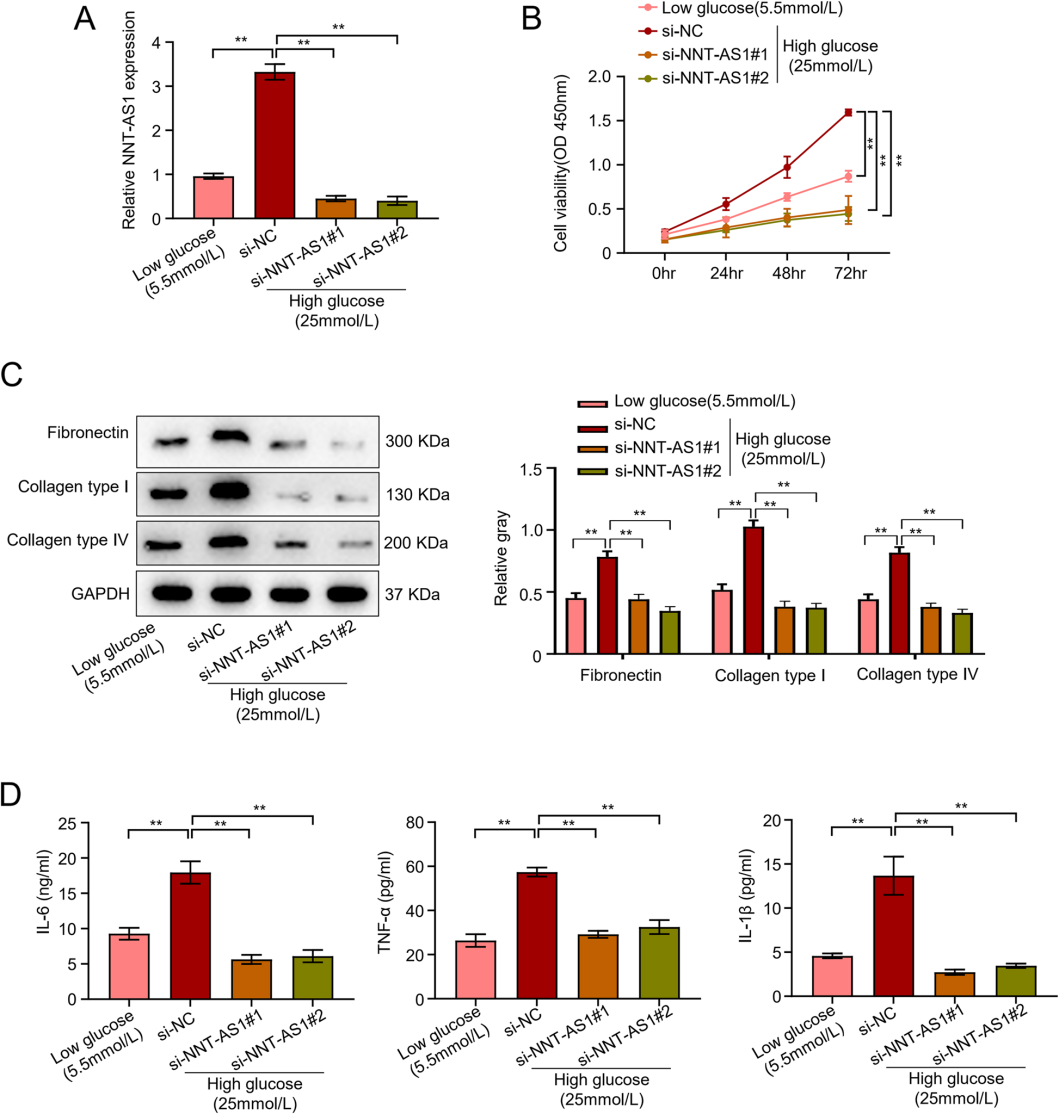
NNT-AS1 regulates HG-induced proliferation, ECM accumulation, and inflammation in mesangial cells. **A** Knocking down NNT-AS1 inhibited the proliferation, migration and invasion of pancreatic cancer cells; qRT-PCR showing NNT-AS1 knockdown in HGMCs (*P* < 0.01), **B** the light absorption values of HGMC cells transfected with small-interfering RNAs to assess the proliferative ability of the HGMCs through CCK8 assay in LG and HG-induced cells (*P* < 0.01), **C** western blot analysis of transfected HGMCs to detect ECM-related proteins including fibronectin (FN), collagen type I (Col-I), and collagen type IV (Col-IV), **D** levels of inflammatory factors including IL-6, IL-1β and TNF-α monitored in different groups of transfected HGMCs by ELISA (*P* < 0.01)

The NNT-AS1 levels in LG groups and HG groups were further validated through a series of experiments. First, the transfection was performed to create HGMC knockouts of NNT-AS1 and fed with a relative concentration of glucose. Following this, NNT-AS1 expression levels were determined by qRT-PCR analysis after transfection with si-NNT-ASI. the qRT-PCR analysis showed significantly increased NNT-AS1 expression levels about 3.5 times higher in HG si-NC (negative control) group than LG si-NC (negative control) group, suggesting that high glucose levels are directly correlated with higher NNT-AS1 expression. Whereas, knocking-down NNT-AS1 with the help of si- NNT-AS1#1 and si- NNT-AS1#2 in HG group completely reversed the effects and showed a considerable decrease in (up to 80%) NNT-AS1 expression with the statistical significance of *P* < 0.01 (Fig. 2A). Cell proliferation was noted by the standard CCK-8 bioassay after measuring the light absorption values of HGMC cells after specific time points. The results showed clear consistency with the previously performed qRT-PCR analysis demonstrating low light absorption values and proliferative ability (up to 45%) of the low glucose (LG) group as compared to high glucose (HG) negative control group. Furthermore, light absorption values of HGMC cells showed a significant drop and decreased proliferation (about 70%) after transfection with si-NNT-AS1#1 and si-NNT-AS1#2 in the HG group. Both small interfering RNAs including si-NNT-AS1#1 and si-NNT-AS1#2 showed a similar trend after transfection (*P* < 0.01, Fig. 2B). Extracellular matrix (ECM) related proteins including collagen type IV and I (Col-IV, Col-I) and fibronectin (FN) were estimated through western blot analysis in transfected HGMC lines. All proteins exhibited significantly elevated levels about 2 times higher in HG si-NC group than the LG group indicating their increased expression in mesangial cells. The results revealed that the silencing of NNT-AS1 with both si-RNAs including si-NNT-AS1#1 and si-NNT-AS1#2 decreased the levels (almost 50%) of ECM related proteins with the statistical significance of *P* < 0.01 (Fig. 2C). In addition to ECM related proteins, inflammatory proteins including IL-1β, TNF-α and IL-6 levels were also monitored in different groups of transfected HGMCs. The validation of the hypothesis that NNT-AS1 acts as a regulator of inflammatory cytokines showed increased cytokine levels (almost 2 times) in HG si-NC group as compared to low glucose (LG) group. However, knocking-down NNT-AS1 with si-NNT-AS1#1 and si-NNT-AS1#2 reversed the effects and demonstrated decreased levels (about 60%) of IL-1β, TNF-α and IL-6 (*P* < 0.01, Fig. 2D).

### NNT-AS1 targets miR-214-5p

**Fig. 3:**
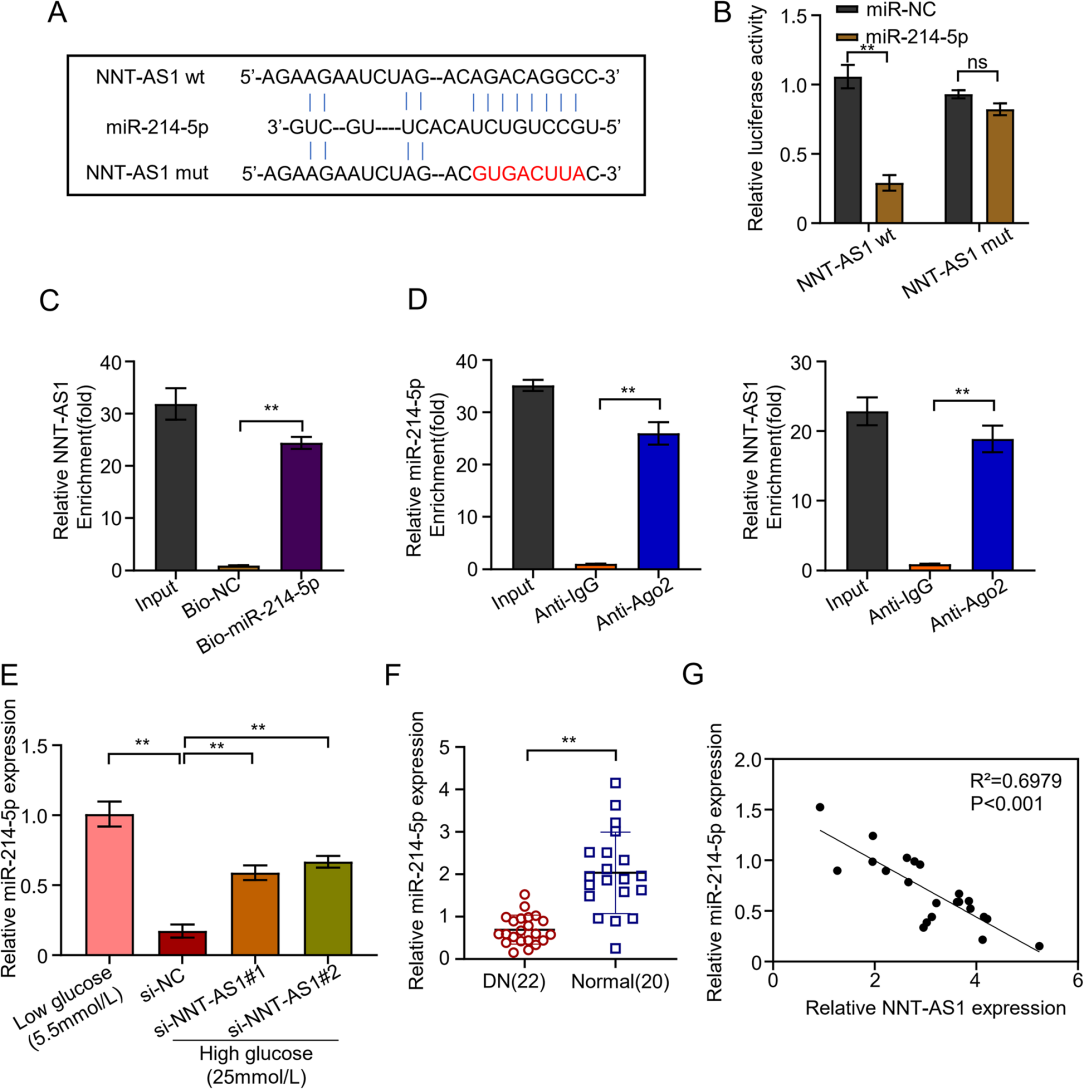
NNT-AS1 targets miR-214-5p. **A** Potential binding elements of miR-214-5p in NNT-AS1 identified through LncBASE database, **B** relative luciferase assay confirming reduced miR-214-5p expression in HGMC lines inoculated with wild-type (WT) and mutant NNT-AS1, **C** RNA pull-down experiment using biotin-labeled NNT-AS1 probe showing more pulled down miR-214-5p (*P* < 0.01) as compared to oligo probe, **D** RNA immunoprecipitation demonstrating more enrichment of NNT-AS1 and miR-214-5p by Ago2 in comparison to IgG group, **E** qRT-PCR analysis showing elevated miR-214-5p expression in the HGMC groups transfected with si-NNT-AS1#1 and si-NNT-AS1#2, **F** the expression level of miR-214-5p in blood samples of 22 patients with DN and 20 normal persons was detected by qRT-PCR, **G** Spearman correlation coefficient showing significant negative correlation between the expression of NNT-AS1 and miR-214-5p

Potential binding elements of miR-214-5p in NNT-AS1 were identified through LncBASE online database (Fig. 3A). The results showed the clear binding site of NNT-AS1 in miR-214-5p elucidating miR-214-5p as a potential target of NNT-AS1. Moreover, the development of NNT-AS1 mutant showed its inability to bind miR-214-5p confirming it as a hindering molecule of miR-214-5p. Following this, relative luciferase assay confirmed reduced miR-214-5p expression almost 73% in HGMC lines inoculated with NNT-ASI wild-type (WT) than miR-NC and NNT-AS1 mutant showed relatively same expression as miR-NC (Fig. 3B). RNA pull-down experiment using biotin-labelled NNT-AS1 probe confirmed that NNT-AS1 interacted with miR-214-5p in HGMC cells and pulled down miR-214-5p significantly (*P* < 0.01) up to 24 times higher than probe (Fig. 3C). Finally, the results from RNA immunoprecipitation demonstrated more enrichment of NNT-AS1 (26 times) and miR-214-5p (18 times) by Ago2 in comparison to IgG group (Fig. 3D). Following these, expression of miR-214-5p was confirmed in transfected HGMC lines to validate if NNT-AS1 directly affects the miR-214-5p expression or not. The results suggested the decreased expression of miR-214-5p (80%) in the si-NC group containing active NNT-AS1. Whereas, the expression of miR-214-5p was considerably higher (about 3 times) (*P* < 0.01) in the HGMC groups transfected with si-NNT-AS1#1 and si-NNT-AS1#2 in which the NNT-AS1 was silenced (Fig. 3E). Similarly, miR-214-5p expression in 22 patients with DN and 20 normal persons was detected by qRT-PCR and the results revealed remarkably decreased miR-214-5p expression (up to 66%) in DN patients as compared to normal individuals confirming NNT-AS1 targets miR-214-5p and statistically significant differences were observed (*P* < 0.01, Fig. 3F). In addition, decreased miR-214-5p expression significantly negative correlated with certain known clinical parameters of DN, including high density lipoprotein (HDL), triglyceride (TG) and Albuminuria (*P* < 0.01, Table 1). NNT-AS1 and miR-214-5p correlation that how they influence each other was further investigated in DN patients and normal healthy subjects. Spearman correlation coefficient demonstrated these two as negatively correlated to each other confirming NNT-AS1 as the regulator of miR-214-5p which deprives miR-214-5p from its normal functioning (*R*^2^ = 0.6979, *P* < 0.01, Fig. 3G).

**Table 1:** Correlation between miR-214-5p and clinical parameters of DN

miR-214-5p	DN patients (22)		
	Mean ± SD	γ	***p***-value
**Age (years)**	46 ± 8.00	0.125	0.581
**eGFR (ml/min/1.73 m**^**2**^**)**	101.70 ± 4.94	0.453	0.034
**BMI (kg/ m**^**2**^**)**	25.97 ± 2.22	−0.383	0.078
**HbA1c(%)**	9.45 ± 0.73	0.051	0.820
**HDL (mg/dL)**	46.25 ± 2.77	0.572	0.005
**TG (mg/dL)**	225.22 ± 16.27	−0.559	0.007
**Albuminuria (mg/days)**	57.46 ± 22.48	−0.676	0.001

*DN* Diabetic nephropathy, *eGFR* Estimated glomerular filtration rate, *BMI* Body mass index, *HbA1c* Glycated hemoglobin, *HDL* High density lipoprotein, *TG* Triglyceride

### The miR-214-5p targets smad4

**Fig. 4:**
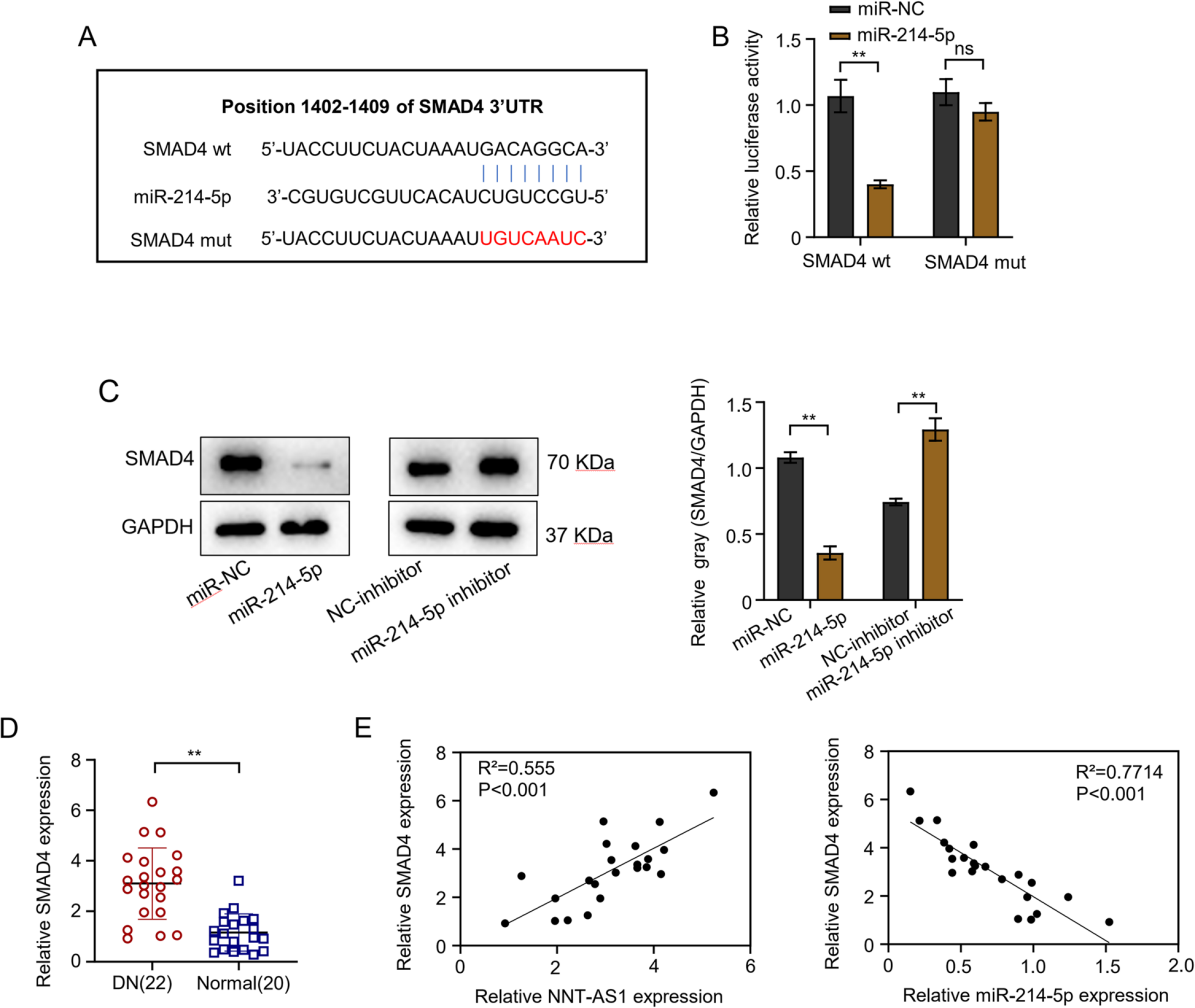
The miR-214-5p targets smad4. **A** TargetScan software showing 3′-UTR site of smad4 as the possible target of miR-214-5p, **B** Results from luciferase reporter assay confirming HGMCs transfected with miR-214-5p inhibited luciferase activity in the cells, **C** the expression levels of smad4 analyzed through western blot analysis in transfected HGMCs transfected with miR-NC, miR-214-5p, NC-inhibitor, and miR-214-5p inhibitor, **D** expression level of smad4 in blood samples of 22 patients with DN and 20 normal persons was detected by qRT-PCR, **E** Spearman correlation coefficient used to analyze the correlation between SMAD4 and NNT-AS1 and miR-214-5p expression in blood samples of 22 patients with DN and 20 normal individuals

TargetScan software predicted the 3′-UTR site of smad4 which might be the possible target of miR-214-5p and luciferase reporter assay was performed for verification (Fig. 4A). The luciferase reporter assay results revealed the reduced luciferase activity (about 60%) in HGMCs cells transfected with miR-214-5p, whereas, the inhibition effect was completely disappeared after the mutation of predicted smad4 3′-UTR binding site (Fig. 4B). Furthermore, the expression levels of smad4 were analyzed through western blot analysis in HGMCs transfected with miR-NC, NC-inhibitor, miR-214-5p and miR-214-5p inhibitor. The results showed decreased expression (68%) of smad4 protein in the miR-214-5p transfected group as compared to the miR-NC group. This indicated the smad4 protein as a potential target of miR-214-5p and decreased its expression. Likewise, as compared to NC-inhibitor group, miR-214-5p inhibitor relatively increased (almost 1.7 times) the smad4 protein expression (Fig. 4C). The qRT-PCR results revealed significantly increased expression (about 3 times) of smad4 in DN patients as compared to normal individuals confirming miR-214-5p targeted the smad4 (*P* < 0.01, Fig. 4D). The correlation between SMAD4, NNT-AS1 and miR-214-5p expression in DN patients and normal individuals was determined by Spearman correlation analysis. Data showed a positive correlation between NNT-AS1 and SMAD4 expression levels (*R*^2^ = 0.555), whereas, a negative correlation was observed between SMAD4 and miR-214-5p expression levels (*R*^2^ = 0.7714) and the differences were statistically significant (*P* < 0.01, Fig. 4E).

### NNT-AS1 is a regulator of Hg-induced proliferation and causes accumulation of ECM and inflammation in mesangial cells through miR-214-5p/smad4 axis

**Fig. 5:**
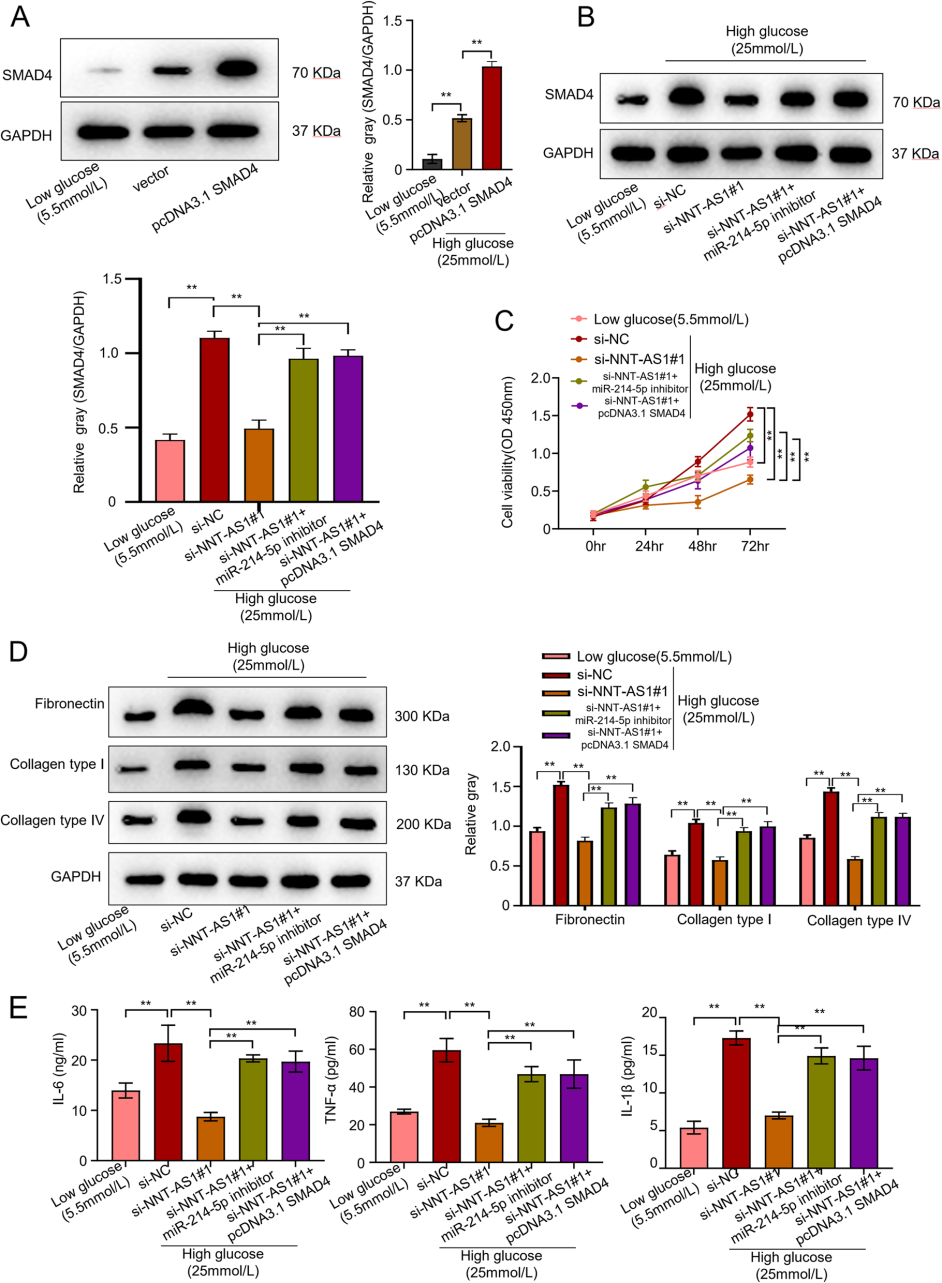
NNT-AS1 regulates Hg-induced proliferation, ECM accumulation, and inflammation in mesangial cells through miR-214-5p/smad4 axis. **A** comparative analysis of smad4 protein in HGMCs through western blot analysis showing smad4 levels in vector, pcDNA-smad4 and LG group, **B** western blot analysis to analyze the correlation between silencing of NNT-AS1 and smad4 in HGMCs divided into certain groups including LG-group, si-NC, si-NNT-AS1#1, si-NNT-AS1#1 + miR-214-5P inhibitor, and si-NNT-AS1#1 + smad4 HG groups, **C** CCK-8 bioassay to evaluate the light absorption values of HGMCs transfected with various variables, **D** western blot analysis of ECM related proteins including fibronectin (FN), collagen type I (Col-I), and collagen type IV (Col-IV) in transfected HGMC lines, **E** ELISA to evaluate the levels of inflammatory-related proteins such as IL-6, IL-1β and TNF-α in different groups of transfected HGMCs

Mechanistic insights into NNT-AS1 functioning were made to analyze how this non-coding RNA influences the human mesangial cells. Early investigations showed the direct influence of NNT-AS1 on miR-214-5p which then influences smad4 and causes proliferation in DN patients. Comparative analysis of smad4 protein through western blot analysis showed that as compared to low glucose (LG) group, smad4 levels were considerably high (up to 5 times) in vector. Whereas, the expression of smad4 was significantly upregulated (about 2 times) in HGMCs transfected with pcDNA-smad4 (Fig. 5A). Furthermore, HGMCs divided into certain groups including LG-group, si-NC, si-NNT-AS1#1, si-NNT-AS1#1 + miR-214-5P inhibitor, and si-NNT-AS1#1 + smad4 induced with HG were subjected to western blot analysis to analyze the correlation between silencing of NNT-AS1 and smad4. The data showed that knocking-down NNT-AS1 decreased the levels (almost 50%) of smad4 protein in cells and si-NC group demonstrated the highest expression (2.7 times) smad4 protein. This is a clear indication that NNT-AS1 work through miR-214-5p/smad4 axis and directly participates in the proliferation of mesangial cells with DN (*P* < 0.01, Fig. 5B). Similarly, CCK-8 bioassay was conducted to evaluate the light absorption values of HGMCs transfected with various variables. The data demonstrated that in comparison to the si-NC group, knocking down of NNT-AS1 reduced the light absorption values (up to 57%) of HGMCs indicating that NNT-AS1 directly participates in the proliferation of mesangial cells. Whereas, the high glucose-induced HGMCs transfected with si-NNT-AS1#1 + miR-214-5p-inhibitor and si-NNT-AS1#1 + smad4 showed a considerable increase in light absorption values (2 times) of the cells when compared with si-NNT-AS#1 (*P* < 0.01, Fig. 5C). ECM related proteins including Col-IV, Col-I and FN were estimated through western blot analysis in transfected HGMC lines. Again, the data showed the results as anticipated and HGMCs with HG in the si-NC group showed considerably elevated levels (almost 1.6 times) of all ECM proteins as compared to LG-group. Whereas in comparison to HGMCs transfected with si-NNT-AS1#1, cells transfected with si-NNT-AS1#1 + miR-214-5p-inhibitor and si-NNT-AS1#1 + smad4 showed a partial increase (up to 1.5 times) in ECM proteins. Thus, the data showed that silencing NNT-AS1 decreases the ECM related proteins (about 50%) which became visibly elevated in the si-NC group (*P* < 0.01, Fig. 5D). Finally, ELISA was conducted to evaluate the IL-1β, IL-6 and TNF-α levels in different groups of transfected HGMCs. The data showed decreased amounts (up to 60%) of IL-1β, IL-6 and TNF-α in the cells with knocked-down NNT-AS1 in comparison to the si-NC group which had active NNT-AS1. Likewise, HGMCs transfected with si-NNT-AS1#1 + miR-214-5p-inhibitor and si-NNT-AS1#1 + smad4 showed a partial increase (almost 2 times) in the IL-1β, IL-6 and TNF-α concentrations (*P* < 0.01, Fig. 5E).

## Discussion

Carcinogenic roles and altered expression profiles of non-coding RNAs are no more hidden truth and have been demonstrated in mounting research reports [24]. Not only can they modulate chromatin structure, act as gene splicing and translation agents, but also sponge several mi-RNAs and influence their role in normal body functions [9]. Likewise, regulatory roles of divergent lncRNAs have been described in diabetic nephropathy, renal fibrosis and metastasis of tumorous cells. For example, PVT1 is a very critical lncRNA associated with DN patients which accumulates ECM and leads to expansion of mesangial cells and knockdown of PVT1 has been associated with the reduction of ECM including type IV collagen (COL4A1), FN, and other related proteins [14]. Likewise, NEAT1 is a lncRNA which shows its elevated expression in hyperglycemia and has been shown to directly interfere AKT/mTOR signalling pathway. Animal models developed with silenced NEAT1 showed reduced ECM related proteins including FN, and COL4A1 [25]. Similarly, a very interesting study demonstrated the role of lncRNA ERBB4-IR in renal fibrosis and its silencing protected the rat models from high albuminuria and inhibiting the development of DN [26]. The same way, NNT-AS1 has been well described to be associated with lung and bladder cancer and other diseases [16–18]. However, none of them illustrates the mechanism of NNT-AS1 in development, progression and disease development of human mesangial cells. This study was designed to illustrate the significance and molecular mechanisms of NNT-AS1 in the regulation of inflammatory proteins, and inflammation of human mesangial cells. Analysis of LncBASE showed upregulated levels of NNT-AS1 in diabetic nephropathy patients which was further confirmed by high glucose induced HGMCs. The data confirmed that diabetic patients have elevated levels of NNT-AS1 which progressively leads to DN. Upregulated levels of NNT-AS1 have been previously observed in certain cancers. For example, poor prognosis and progression of osteosarcoma has been associated with NNT-AS1 [27]. Similarly, a study reported overexpressed NNT-AS1 in ovarian cancer and lead to its progression and development [28].

Moreover, several studies showed that lncRNAs participate in cancer development by sponging certain micro-RNAs. In this study also, experiments showed that NNT-AS1 does so by binding to miR-214-5p which gets absorbed by this non-coding RNA and thus cannot perform its normal functioning. This is a well-explored mechanism where NNT-AS1 has shown to be a sponge of several microRNAs. For example, NNT-AS1 causes proliferation and increases the invasiveness of tumorous cells by regulating miR-129-5p [29]. Similarly, NNT-AS1 acts as a sponge for miR-363 and causes the metastasis in hepatocellular carcinoma [30]. Furthermore, NNT-AS1 has been studied to modulate miR-485 and acts as an oncogene in cholangiocarcinoma [31]. This study has also proved NNT-AS1 a sponge for miR-214-5p where it was completely masked by NNT-AS1 and could not perform its regulatory functions. Experiments showed that knocking-down NNT-AS1 increased the miR-214-5p expression in the cells. Moreover, it was shown that NNT-AS1 acts through regulating miR-214-5p/smad4 axis. Smad4 is a tumour suppressor gene and its mutation or control indirectly disturbs the TGFβ signalling pathway. The gene is responsible for encoding a transcription-factor and ultimately stimulates and represses target gene expression. Smad4 has been evaluated for its tumor suppressive role in colorectal cancer, colon cancer and its tumor-progressive role in hepatocellular carcinoma [32–34].

## Conclusion

In this study, data showed that overexpressing miR-214-5p decreased the expression of smad4 whereas, upregulated levels of NNT-AS1 directly influenced the pathway. In conclusion, the study showed the effects of overexpressed NNT-AS1 in proliferation, ECM accumulation, and inflammation of HGMCs. Also, the results manifested that knocking-down NNT-AS1 can significantly decrease the inflammation and progression of DN and hence can be used as a potential therapeutic target.

## Supplementary Information

**Additional file 1.**

## Data Availability

The datasets during and/or analyzed during the current study available from the corresponding author on reasonable request.

## Abbreviations

NNT-AS1: LncRNA NNT-AS1
DN: Diabetic nephropathy
HGMCs: Human glomerular mesangial cells
ECM: Extracellular matrix
HG: High glucose
LG: Low glucose
RIP: RNA immunoprecipitation
Col-IV, Col-I: Collagen type IV and I
FN: Fibronectin
COL4A1: Type IV collagen
