# Supplementary material for: LncRNA NNT-AS1 regulates proliferation, ECM accumulation and inflammation of human mesangial cells induced by high glucose through miR-214-5p/smad4
Source: BMC Nephrol. 2021 Nov 6;22:368. doi: 10.1186/s12882-021-02580-y (PMC8572446; doi:10.1186/s12882-021-02580-y)
Supplement: Supplementary file 1 — Additional file 1. [file 12882_2021_2580_MOESM1_ESM.pptx]

## Slide 1
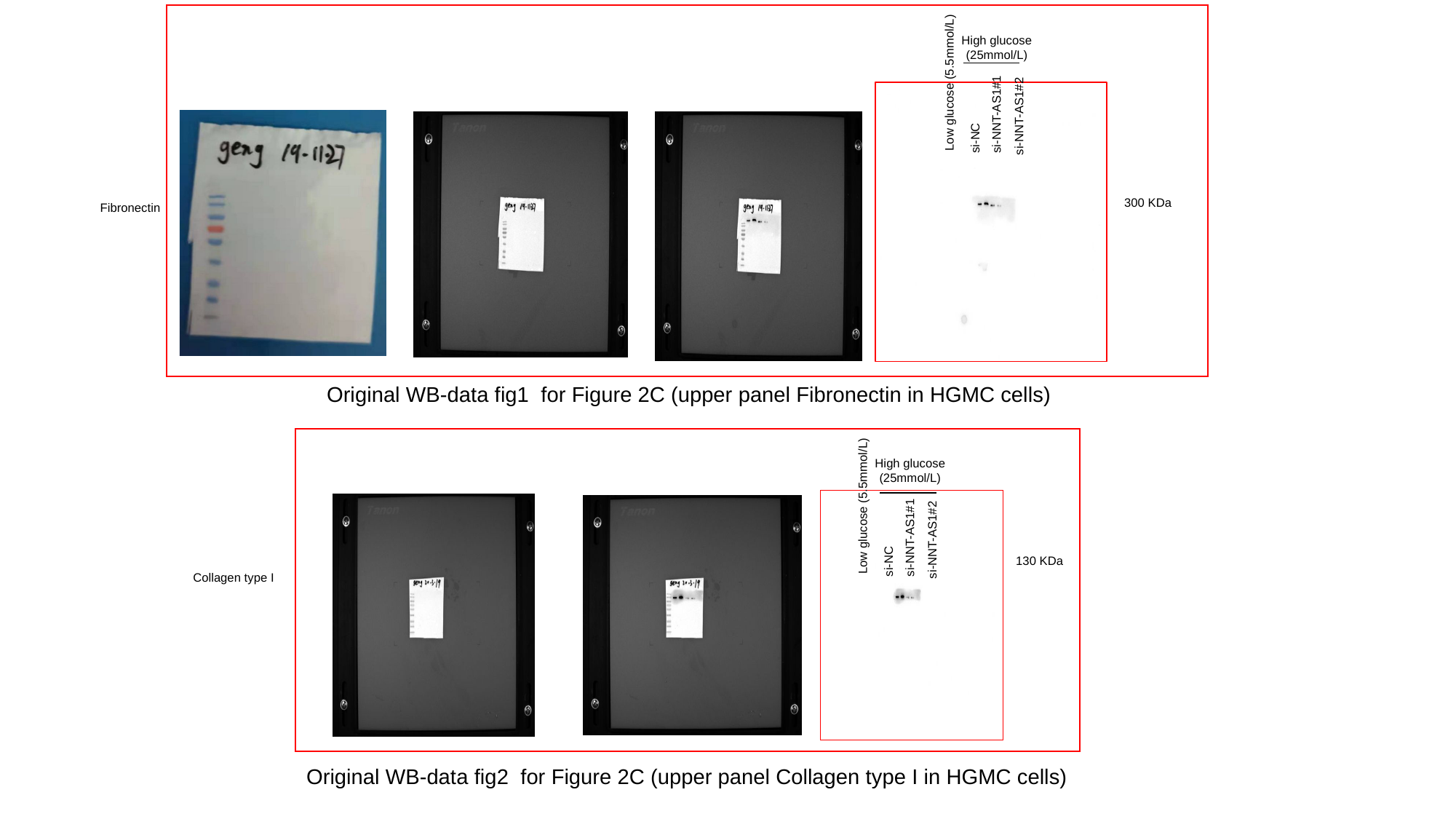

High glucose
(25mmol/L)
Low glucose (5.5mmol/L)
si-NNT-AS1#1
si-NNT-AS1#2
si-NC
300 KDa
Fibronectin
Original WB-data fig1 for Figure 2C (upper panel Fibronectin in HGMC cells)
High glucose
(25mmol/L)
Low glucose (5.5mmol/L)
si-NNT-AS1#1
si-NNT-AS1#2
130 KDa
si-NC
Collagen type I
Original WB-data fig2 for Figure 2C (upper panel Collagen type I in HGMC cells)

## Slide 2
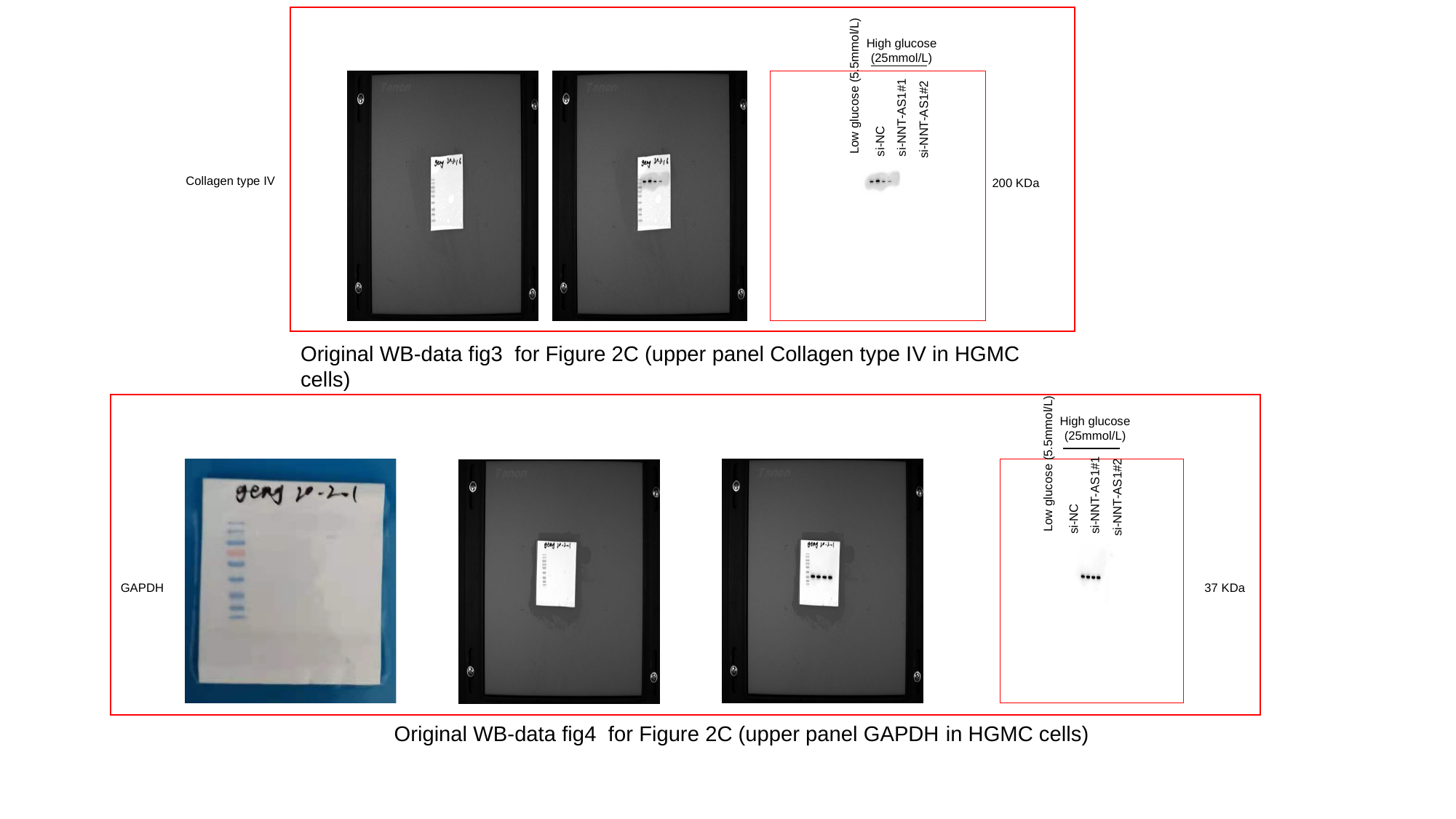

High glucose
(25mmol/L)
Low glucose (5.5mmol/L)
si-NNT-AS1#1
si-NNT-AS1#2
si-NC
Collagen type IV
200 KDa
Original WB-data fig3 for Figure 2C (upper panel Collagen type IV in HGMC cells)
High glucose
(25mmol/L)
Low glucose (5.5mmol/L)
si-NNT-AS1#1
si-NNT-AS1#2
si-NC
GAPDH
37 KDa
Original WB-data fig4 for Figure 2C (upper panel GAPDH in HGMC cells)

## Slide 3
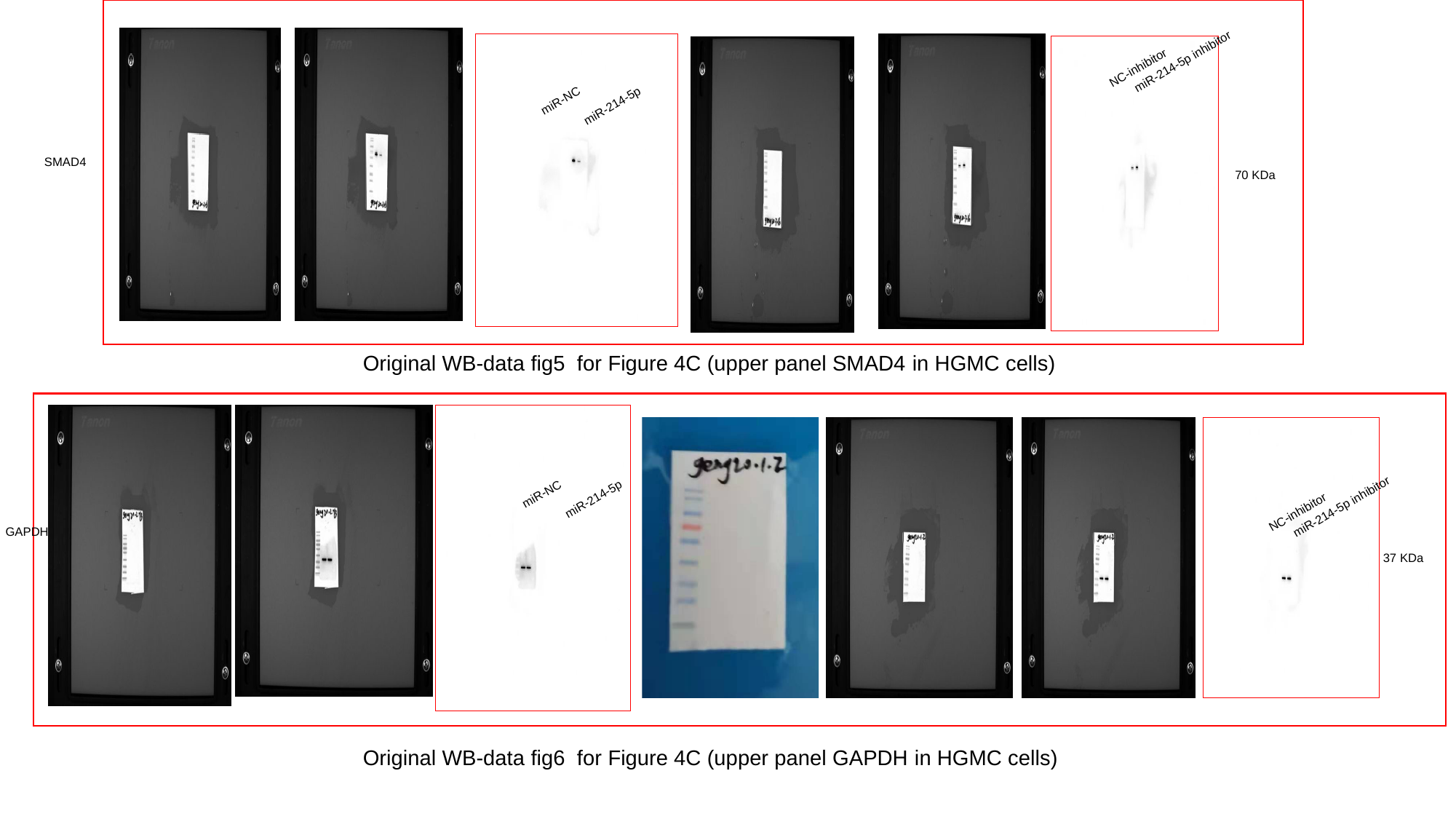

miR-214-5p inhibitor
NC-inhibitor
miR-NC
miR-214-5p
SMAD4
70 KDa
Original WB-data fig5 for Figure 4C (upper panel SMAD4 in HGMC cells)
miR-NC
miR-214-5p
miR-214-5p inhibitor
NC-inhibitor
GAPDH
37 KDa
Original WB-data fig6 for Figure 4C (upper panel GAPDH in HGMC cells)

## Slide 4
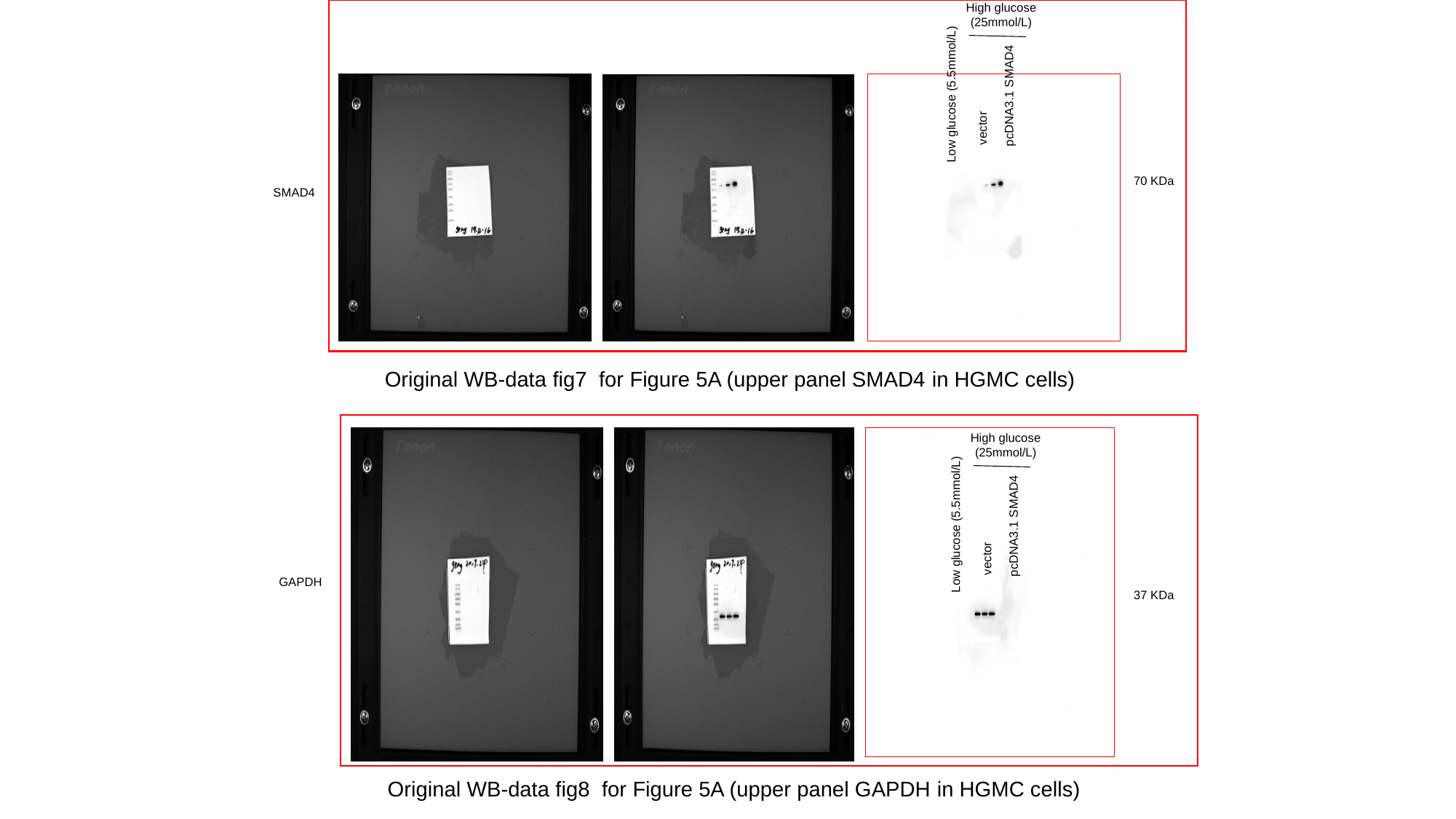

High glucose
(25mmol/L)
Low glucose (5.5mmol/L)
pcDNA3.1 SMAD4
vector
70 KDa
SMAD4
Original WB-data fig7 for Figure 5A (upper panel SMAD4 in HGMC cells)
High glucose
(25mmol/L)
Low glucose (5.5mmol/L)
pcDNA3.1 SMAD4
vector
GAPDH
37 KDa
Original WB-data fig8 for Figure 5A (upper panel GAPDH in HGMC cells)

## Slide 5
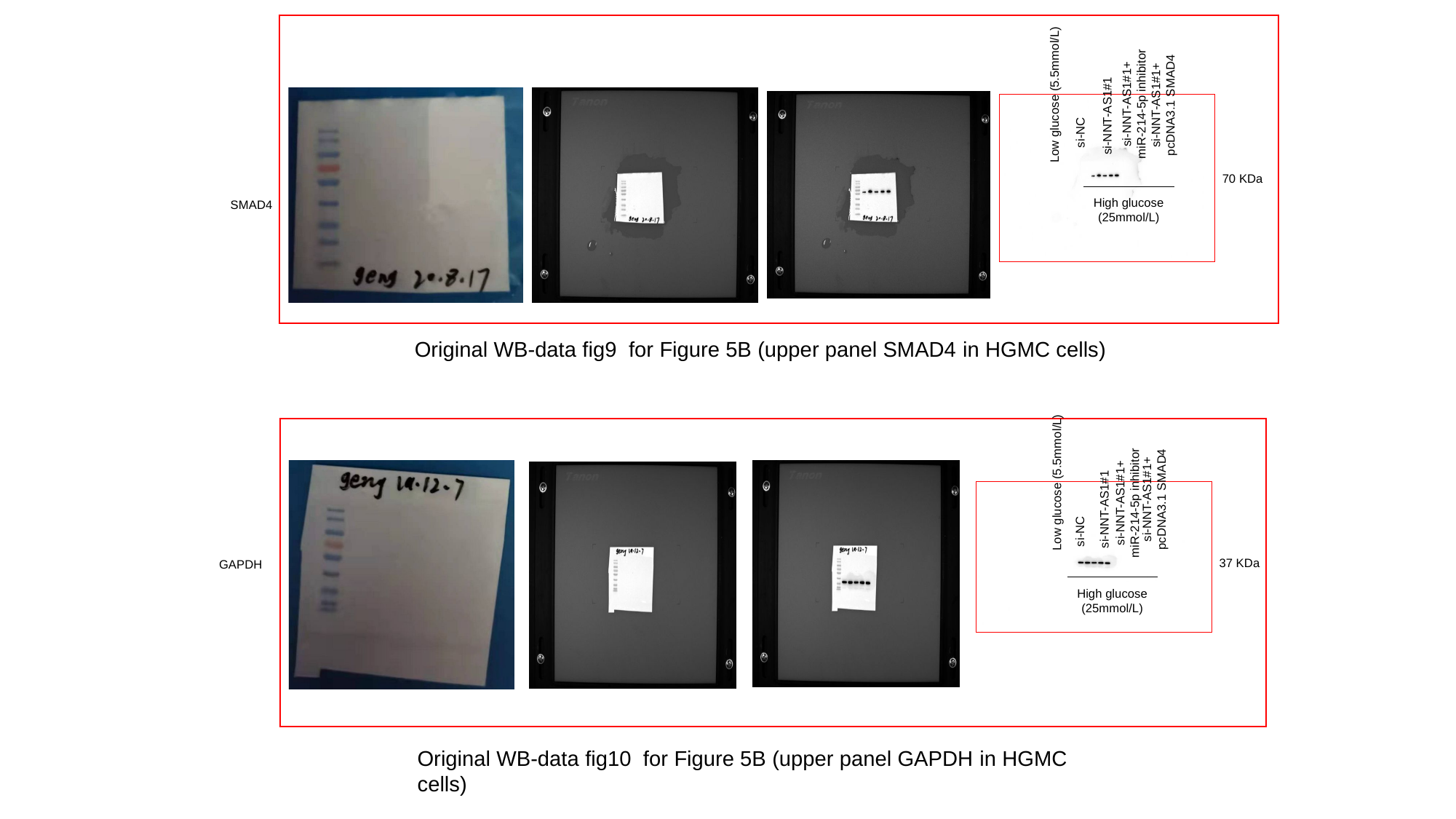

Low glucose (5.5mmol/L)
si-NNT-AS1#1+
miR-214-5p inhibitor
si-NNT-AS1#1+
pcDNA3.1 SMAD4
si-NNT-AS1#1
si-NC
70 KDa
High glucose
(25mmol/L)
SMAD4
Original WB-data fig9 for Figure 5B (upper panel SMAD4 in HGMC cells)
Low glucose (5.5mmol/L)
si-NNT-AS1#1+
pcDNA3.1 SMAD4
si-NNT-AS1#1+
miR-214-5p inhibitor
si-NNT-AS1#1
si-NC
37 KDa
GAPDH
High glucose
(25mmol/L)
Original WB-data fig10 for Figure 5B (upper panel GAPDH in HGMC cells)

## Slide 6
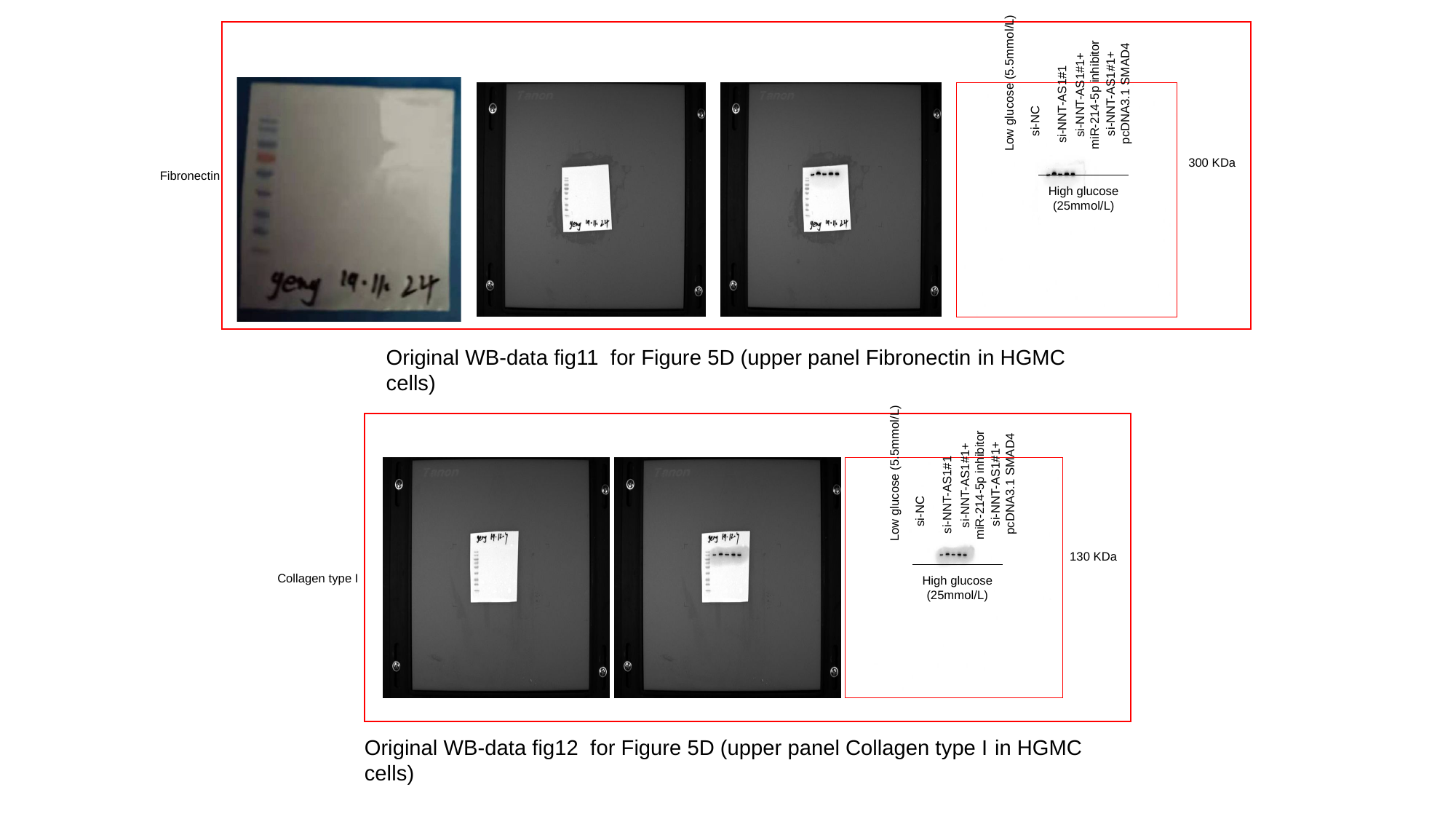

Low glucose (5.5mmol/L)
si-NNT-AS1#1+
pcDNA3.1 SMAD4
si-NNT-AS1#1+
miR-214-5p inhibitor
si-NNT-AS1#1
si-NC
300 KDa
Fibronectin
High glucose
(25mmol/L)
Original WB-data fig11 for Figure 5D (upper panel Fibronectin in HGMC cells)
Low glucose (5.5mmol/L)
si-NNT-AS1#1+
pcDNA3.1 SMAD4
si-NNT-AS1#1+
miR-214-5p inhibitor
si-NNT-AS1#1
si-NC
130 KDa
Collagen type I
High glucose
(25mmol/L)
Original WB-data fig12 for Figure 5D (upper panel Collagen type I in HGMC cells)

## Slide 7
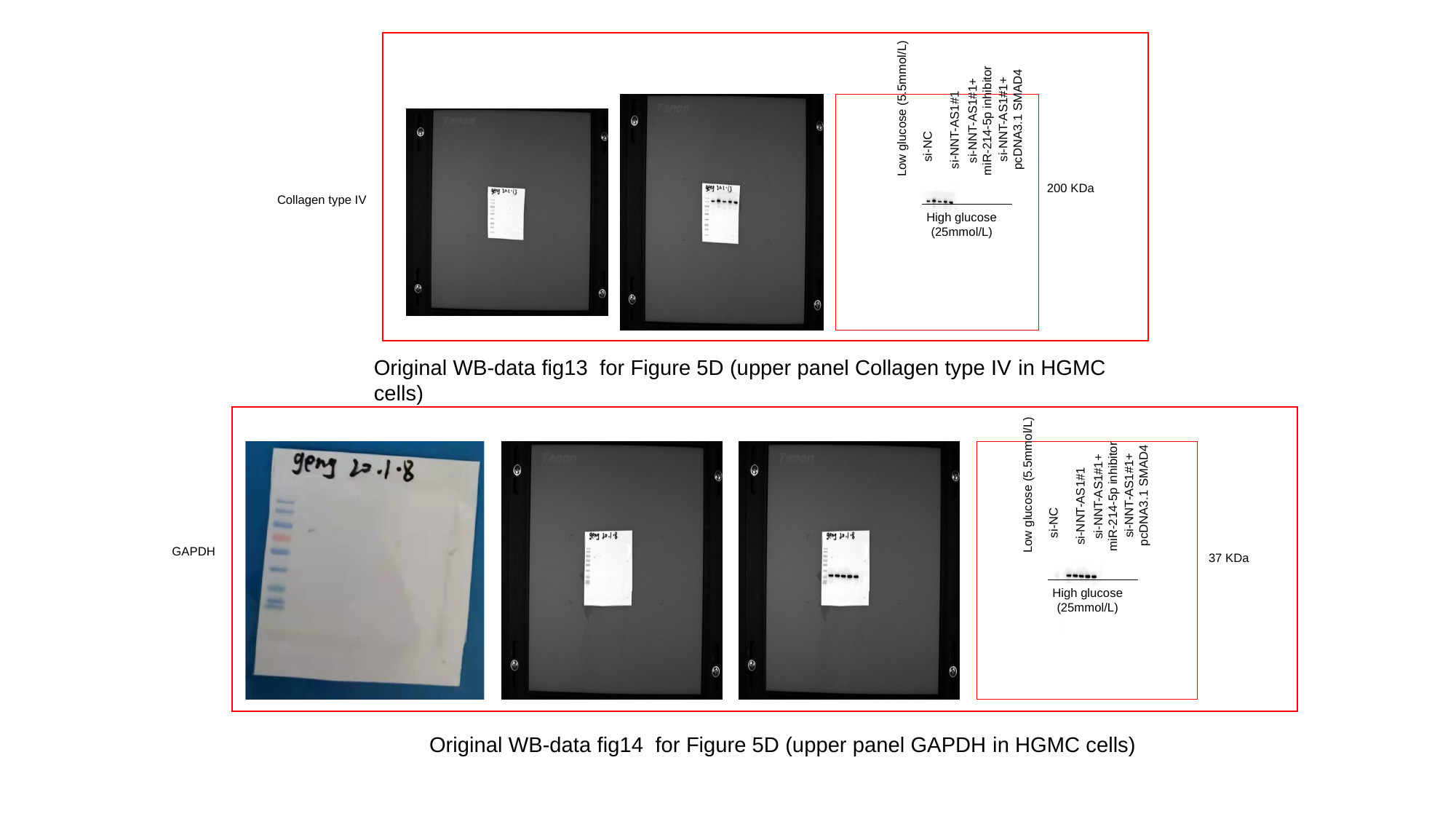

Low glucose (5.5mmol/L)
si-NNT-AS1#1+
pcDNA3.1 SMAD4
si-NNT-AS1#1+
miR-214-5p inhibitor
si-NNT-AS1#1
si-NC
200 KDa
Collagen type IV
High glucose
(25mmol/L)
Original WB-data fig13 for Figure 5D (upper panel Collagen type IV in HGMC cells)
Low glucose (5.5mmol/L)
si-NNT-AS1#1+
pcDNA3.1 SMAD4
si-NNT-AS1#1+
miR-214-5p inhibitor
si-NNT-AS1#1
si-NC
GAPDH
37 KDa
High glucose
(25mmol/L)
Original WB-data fig14 for Figure 5D (upper panel GAPDH in HGMC cells)
